# Validation of a patient-specific finite element analysis framework for identification of growing rod-failure regions in early onset scoliosis patients

**DOI:** 10.1007/s43390-024-00846-7

**Published:** 2024-03-27

**Authors:** Daksh Jayaswal, Manoj Kodigudla, Amey Kelkar, Vijay Goel, Vivek Palepu

**Affiliations:** 1 Department of Bioengineering and Orthopaedic Surgery, Engineering Center for Orthopaedic Research Excellence (E-CORE), University of Toledo, 2801 West Bancroft Street, Toledo, OH 43606 USA; 2 Division of Applied Mechanics, Office of Science and Engineering Laboratories, Center for Devices and Radiological Health, U.S. Food and Drug Administration, 10903 New Hampshire Avenue, Building WO 62-2225, Silver Spring, MD 20993 USA

**Keywords:** Biomechanics, Growing rods, Early onset scoliosis, Finite element analysis, Patient-specific modeling, Rod failures

## Abstract

**Purpose:**

Growing rods are the gold-standard for treatment of early onset scoliosis (EOS). However, these implanted rods experience frequent fractures, requiring additional surgery. A recent study by the U.S. Food and Drug Administration (FDA) identified four common rod fracture locations. Leveraging this data, Agarwal et al. were able to correlate these fractures to high-stress regions using a novel finite element analysis (FEA) framework for one patient. The current study aims to further validate this framework through FEA modeling extended to multiple patients.

**Methods:**

Three patient-specific FEA models were developed to match the pre-operative patient data taken from both registry and biplanar radiographs. The surgical procedure was then simulated to match the post-operative deformity. Body weight and flexion bending (1 Nm) loads were then applied and the output stress data on the rods were analyzed.

**Results:**

Radiographic data showed fracture locations at the mid-construct, adjacent to the distal and tandem connector across the patients. Stress analysis from the FEA showed these failure locations matched local high-stress regions for all fractures observed. These results qualitatively validate the efficacy of the FEA framework by showing a decent correlation between localized high-stress regions and the actual fracture sites in the patients.

**Conclusions:**

This patient-specific, in-silico framework has huge potential to be used as a surgical tool to predict sites prone to fracture in growing rod implants. This prospective information would therefore be vital for surgical planning, besides helping optimize implant design for reducing rod failures.

## Introduction

Treatment of excessive deformity (coronal Cobb angle > 40°) in early onset scoliosis (EOS) has always been challenging due to concurrent management of progressive adolescent growth along with correction of the spinal deformity in these young patients [1, 2]. These growth-friendly surgical techniques can be broadly classified as distraction-based, guided growth, or compression-based techniques [3]. Common examples of these include traditional growing rods, magnetically controlled growing rods, SHILLA growth guidance technique, and, more recently, anterior vertebral body tethers [2, 3]. Barring the latter, a common complication across these techniques is rod fractures. Multiple studies have reported a high incidence (> 25%) of rod fractures endemic to the most used traditional and magnetic growing rod techniques [4–9]. Given the high usage and significant failure incidence of these rods in EOS, research focused on prospective identification of potential failure-prone regions in rods might facilitate improved implant design as well as surgical planning.

Finite element analysis (FEA) is a commonly used tool for performing such evaluations in medical devices, along with traditional benchtop testing options [10, 11]. FEA helps overcome problems inherent to physical experiments for evaluating EOS therapies, i.e., limited availability of relevant scoliotic cadavers, variability of deformity manifestation, and the limited accuracy of stress measurements on implanted constructs [12]. Furthermore, with the advent of patient-specific FEA, the research can be highly specialized/focused and generate meaningful data when compared to a more generalized approach [13]. For example, a study by Henao et al. showed how a patient-specific FEA successfully reproduced the biomechanics of neurological injury during scoliosis correction maneuvers when compared to clinical cases with and without intra-operative neurological complications [14]. In the future, modeling frameworks like these could be used as a tool to aid pre-operative surgical planning. Specifically, the biomechanical data obtained from patient-specific FEA can be applied to simulate different spinal disorders and thus lead to optimization of surgical planning.

In the field of scoliosis, current literature using patient-specific FEA is focused on two specific areas. In the first area, studies focus on validating the modeling framework by quantifying the variation in modeling predictions when compared to clinical outcomes (i.e., Cobb angle correction, kyphosis correction, and spinal height change) [15, 16]. For example, a study conducted by Little and Adam showed low variation in the anatomical measurements of spinal parameters (such as Cobb angle, kyphosis, lordosis, thoracic spinal heights, etc.) across FEA model predictions compared with the mean intra-observer variability for three patients [15]. The second area focuses on the same clinical outcomes but with different correction maneuvers and different instrumentation strategies [17–21]. For example, a recent study by Wang et al. explored the effect of various configurations of rod contouring on three-dimensional spinal correction. The key outcome to evaluate different configurations was to understand their effect on the bone-screw forces stemming from screw pull-out; a known complication in adolescent idiopathic scoliosis surgeries [18].

Building on this work, Agarwal et al. developed a novel patient-specific computational modeling framework and correlated high-stress regions on traditional growing rods with clinical fracture-prone locations for a single patient [22]. This FEA model was developed using clinical registry data obtained as part of a study conducted by the U.S. Food and Drug Administration (FDA) on retrieved failed traditional growing rods across 36 patients [23]. The results of this study verified the proof-of-concept modeling framework, with two of the three rod fracture-prone regions (high-stress locations) matching the retrieval data. The study, however, was limited in that data from a single-patient FEA model. The results of the previous study were compared against broad conclusions from the previous retrieval analysis data [22, 23]. The aim of the current study is to extend this verified framework to multiple EOS patients with patient-specific rod-failure location data that aids in understanding the growing rod failures from a biomechanical perspective. Therefore, the objective of this study is to develop three patient-specific finite element models, simulate the traditional growing rod surgery, and validate the high-stress regions on growing rods with respect to the clinical rod fracture locations. This information about the failure-prone regions on implants would be valuable to the surgeons/end users to supplement surgical planning. Furthermore, this biomechanical analysis may aid manufacturers in device design and development, to optimize implant design for reducing rod failures.

## Methods

This study leverages the existing computational modeling framework to identify and validate high-stress regions on traditional growing rods against prior retrieval analysis fracture data [22]. In the current study, the FEA modeling framework outlined in the sections below is applied to three patients, simulating traditional growing rod surgery, to compare rod-stress data to their respective rod-failure data obtained from the clinical registry. The hypothesis is that the clinical fracture locations will match the high-stress locations from the FEA models.

### Pre-operative scoliotic FE model development

Three patient-specific FEA models of the thoracolumbar spine (T1-S1) were developed to match the pre-operative (pre-op) scoliosis curve to corresponding patient registry (Growing Spine Study Group, San Diego, CA) and biplanar radiographs, as described previously [22]. In brief, a healthy pediatric spine FEA model was modified using a custom MATLAB script (MATLAB Inc., Natick, MA) to induce patient-specific biplanar deformity [8, 9, 22]. Input data for necessary parameters like Cobb angle, thoracic kyphosis, lumbar lordosis, and the spinal height (T1-S1) were measured using Surgimap software (Surgimap, Nemaris Inc., New York, NY) from the patient radiographs (Table 1). The final pre-op parameters for the FEA models were within ± 5° of the radiographic measurements [24, 25].

**Table 1:** Patient characteristics at pre-operative time point. Patient data (gender, age, and weight) and spinal parameters (Cobb angle, kyphosis, and lordosis) for each of the three patients

Patient parameters	Patient 1	Patient 2	Patient 3
Gender	Male	Male	Female
Age (years)	10.2	2.8	7.0
Weight (kg)	28.5	11.4	24.9
Major Cobb angle (°)	90	72	76
Kyphosis (°)	25	64	49
Lordosis (°)	6	−55	−52

### Post-operative scoliotic FE model development

Following the creation of the pre-operative FEA models, patient-specific instrumentation was created using SolidWorks (Dassault Systèmes, SolidWorks Corporation, Waltham, MA, USA) for the three FEA models. Specific data regarding location, material, and geometry of the implants were obtained from post-operative patient radiographs and registry data (Table 2). All designed constructs comprised dual traditional growing rod constructs; multiple tandem connectors and pedicle screws customized for each patient. The instrumentation was then implanted into the pre-operative model. An element size of 0.5 mm was chosen based on the prior mesh convergence study [22]. Material properties, constitutive laws, and element types are described in the previous study [22].

**Table 2:** Patient characteristics at intra-operative time point. Detailed data regarding implants used and their respective locations. Additionally, surgical correction data defining location and magnitude of rod distraction

Surgical inputs	Patient 1	Patient 2	Patient 3
*Implant details*			
Rod material	Cobalt chrome	Cobalt chrome	Titanium
Rod diameter	4.5 mm	3.5 mm	4.5 mm
Proximal bilateral screws (location)	T3, T4	T1, T2	T2, T3
Proximal crosslink (location)	Between T3 and T4	Below T2	Between T2 and T3
Distal bilateral screws (location)	L3, L4	T11, T12	L2, L3
Distal crosslink (location)	Between L3 and L4	Between T11 and T12	Between L2 and L3
Number of tandem connectors	2	2	2
*Surgical correction*			
Applied distraction (location)	Top left rod	Both top rods	Both top rods
Applied distraction (distance)	35.0 mm	6.5 mm	10.0 mm

Next, the implanted pre-op patient-specific scoliotic model was modified to simulate the surgical procedure and thus match the post-op scoliotic curve parameters. This correction was done to match the sagittal contour of rods to the post-op radiographs of the patient, and to obtain the stresses generated on the rods (simulation of rod attachment) [19]. An iterative correction process (model calibration) was done by controlling the magnitude of applied distraction in ABAQUS to each of the three FEA models to match post-operative parameters (Table 3). The final post-op scoliotic curve parameters for the spinal model were within ± 5° of the radiographic measurements except for the kyphosis parameter of patients 1 and 3 [24, 25]. For the aforementioned patients, there was a computational limitation where we could not achieve the necessary kyphosis angle without having an adverse effect on the coronal Cobb angles. However, the primary parameter for quantification of scoliotic deformity is the coronal Cobb angle which was within ± 5°. Furthermore, in patients 2 and 3, we have chosen to use pedicle screws as our proximal anchors in place of laminar or cranial hooks. This choice was based on two reasons. First, our framework did not model any ribcage required for simulation of these devices which would increase the complexity and computational expense. Furthermore, a few clinical studies investigated the effect of using pedicle screws versus proximal hooks, and have shown no significant differences, rather suggesting the use of pedicle screws where possible [26, 27].

**Table 3:** Comparison of post-operative patient parameters with FEA model counterparts. Comparison of coronal (Cobb angle) and sagittal deformity (kyphosis, lordosis) quantified across the three patients. The radiographic calculations were carried out using Surgimap and were compared to the FEA model data

Patient angles	Patient 1				Patient 2				Patient 3			
	Pre-operative data		Post-operative data		Pre-operative data		Post-operative data		Pre-operative data		Post-operative data	
	Surgimap	FEA model	Surgimap	FEA model	Surgimap	FEA model	Surgimap	FEA model	Surgimap	FEA model	Surgimap	FEA model
Cobb Angle (°)	89.8	88.3	29.7	31.6	72.5	72.5	18.7	20.7	76.1	71.7	53.8	49.6
Kyphosis (°)	22.6	18.8	29.2	14.5	64.9	64.3	33.9	35.1	49.9	46.0	19.0	28.7
Lordosis (°)	6.0	6.5	34.7	36.5	54.6	54.5	27.7	30.1	51.9	51.2	48.0	43.7

### Contact and loading conditions

**Fig. 1:**
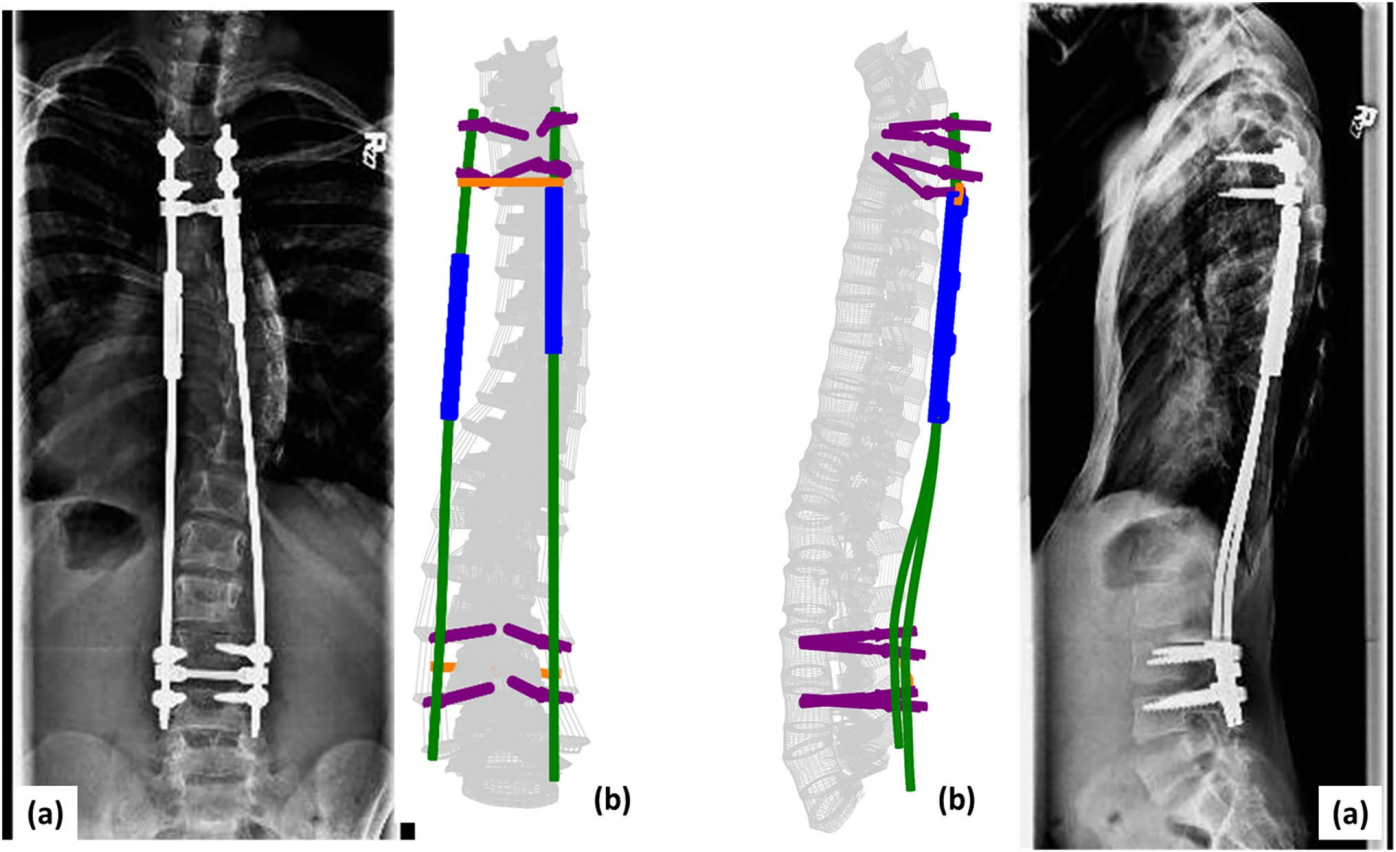
Comparison of the patient-specific finite element model with the patient radiographs for patient 1. **a** The radiographic image and **b** patient-specific finite element model counterparts are shown for coronal (left) and sagittal profiles (right), respectively

**Fig. 2:**
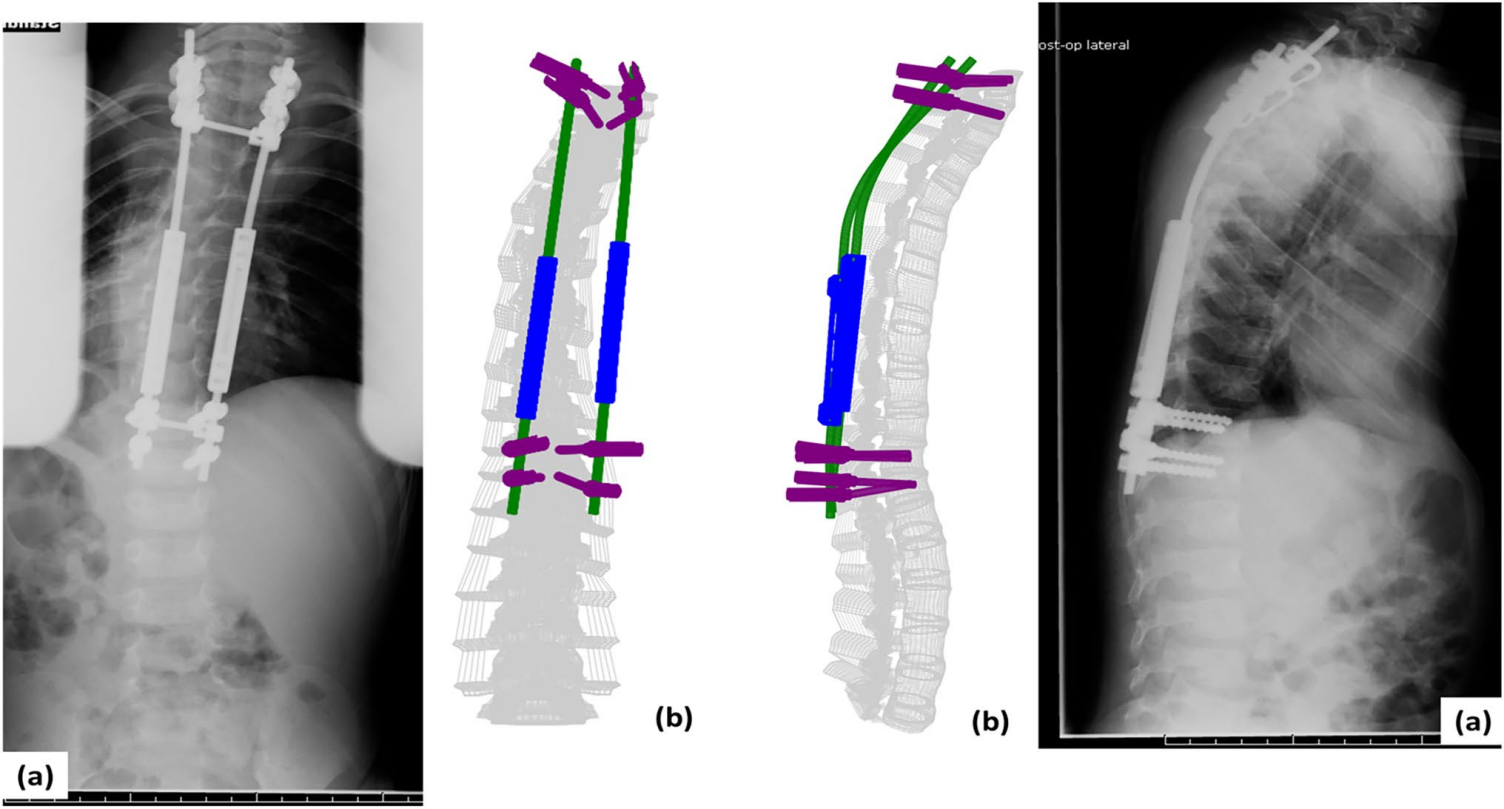
Comparison of the patient-specific finite element model with the patient radiographs for patient 2. **a** The radiographic image and **b** patient-specific finite element model counterparts are shown for coronal (left) and sagittal profiles (right), respectively

**Fig. 3:**
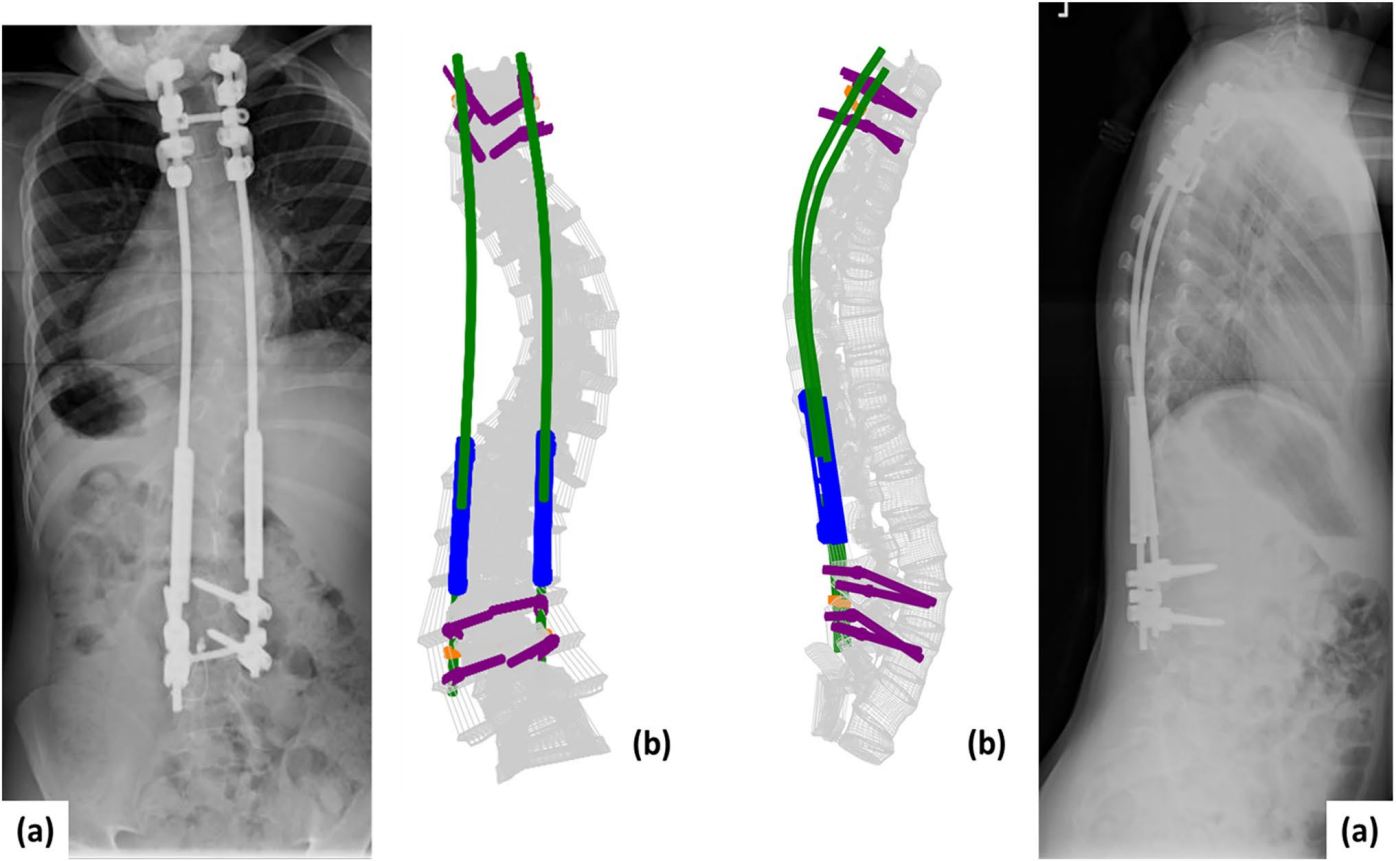
Comparison of the patient-specific finite element model with the patient radiographs for patient 3. **a** The radiographic image and **b** patient-specific finite element model counterparts are shown for coronal (left) and sagittal profiles (right), respectively

For the final step, the patient-specific spinal instrumentation was implanted into the surgically corrected spinal model (Figs. 1, 2, 3). This was followed by application of bilateral longitudinal distraction, application of weight-specific follower load, and application of a 1 Nm flexion moment at the T1 vertebra to simulate worst-case bending motion [22, 23, 28]. The interactions between different interfaces of the FEA model are as listed in similar studies published previously [8, 9, 22]. The inferior endplate (base) of the S1 vertebra was fixed in all directions during all steps.

### Qualitative validation

Spatial distribution of stresses was recorded on the rods after (1) surgical correction and application of distraction forces and follower loads, and (2) following 1 Nm of flexion bending. Next, areas of high-stress concentrations (first principal stress) were identified and compared directly to the corresponding rod-failure locations obtained from the patient-specific radiographs obtained from the retrieval database. The first principal stress was chosen as the preferred stress parameter as it helps in understanding the maximum tensile stress induced in the part due to the loading conditions [29]. This was based on evidence from Hill et al., which revealed that the failure mechanism was due to repeated flexion motion with crack initiation on the posterior side of the rod, causing stresses to be tensile in nature [23].

### Quantitative data evaluation

The study also quantified the location and magnitude of highest principal stress markers that were extracted from the regions on the rods where the respective rod-failure locations were identified. The location of the maximum stress in the FEA model was calculated with respect to the bottom of the nearest tandem connector, as this was the landmark used to calculate the analogous failure location in the clinical radiographs. This location and stress magnitude were compared to the clinical data from radiographs and the global stress maxima, respectively.

## Results

**Fig. 4:**
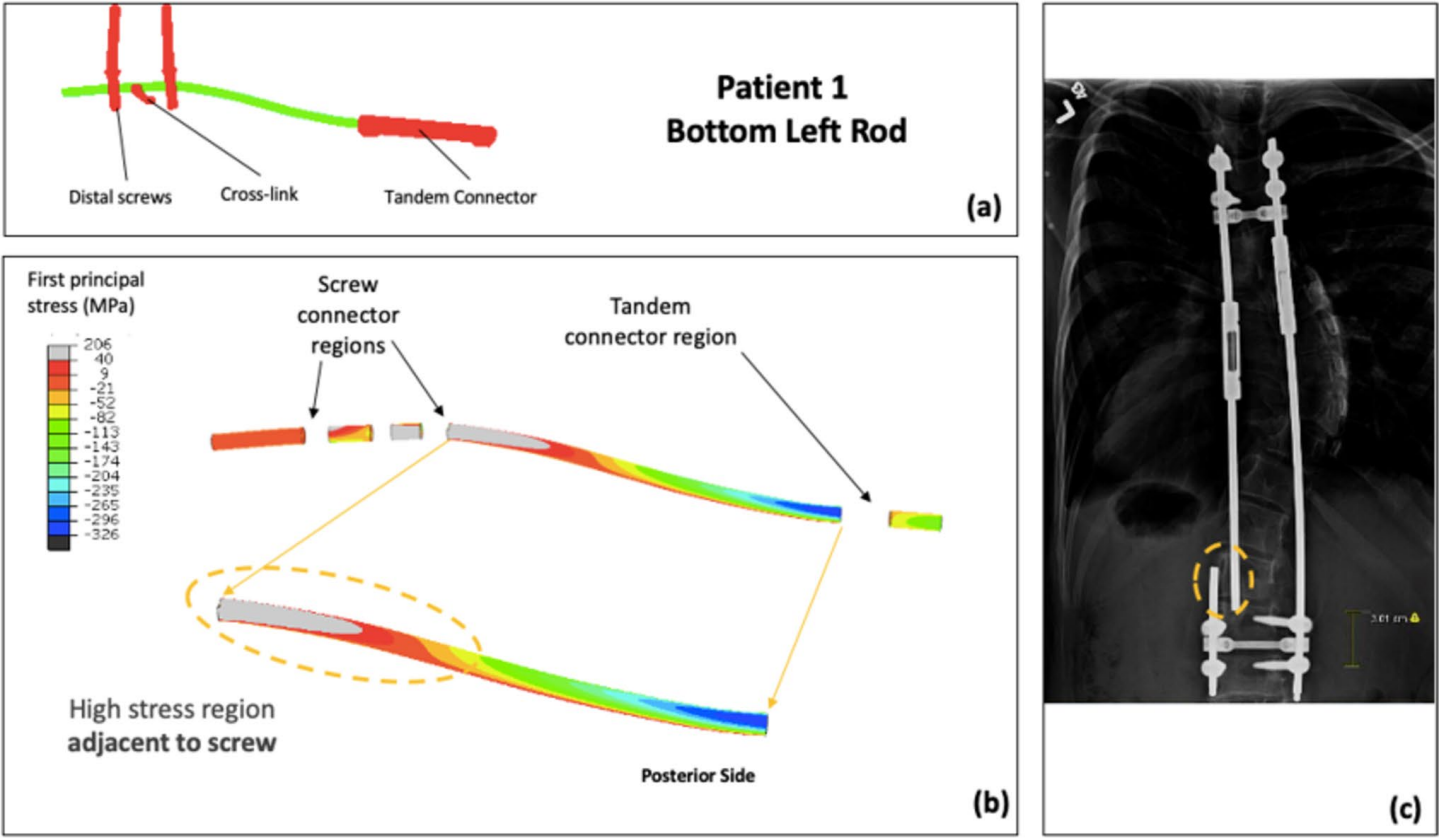
Patient 1 FEA stress distribution plot of bottom left rod (concave side) compared to the corresponding rod-failure location obtained from retrieval analysis data. **a** FE construct showing bottom left rod with tandem connector and screws that were used as part of the traditional growing rod construct simulated in the patient-specific FEA model. **b** Stress distribution on bottom left rod (excluding interacting surfaces). **c** Corresponding patient radiographic image taken from retrieval data to show clinical rod-failure location (encircled) near mid-construct region

**Fig. 5:**
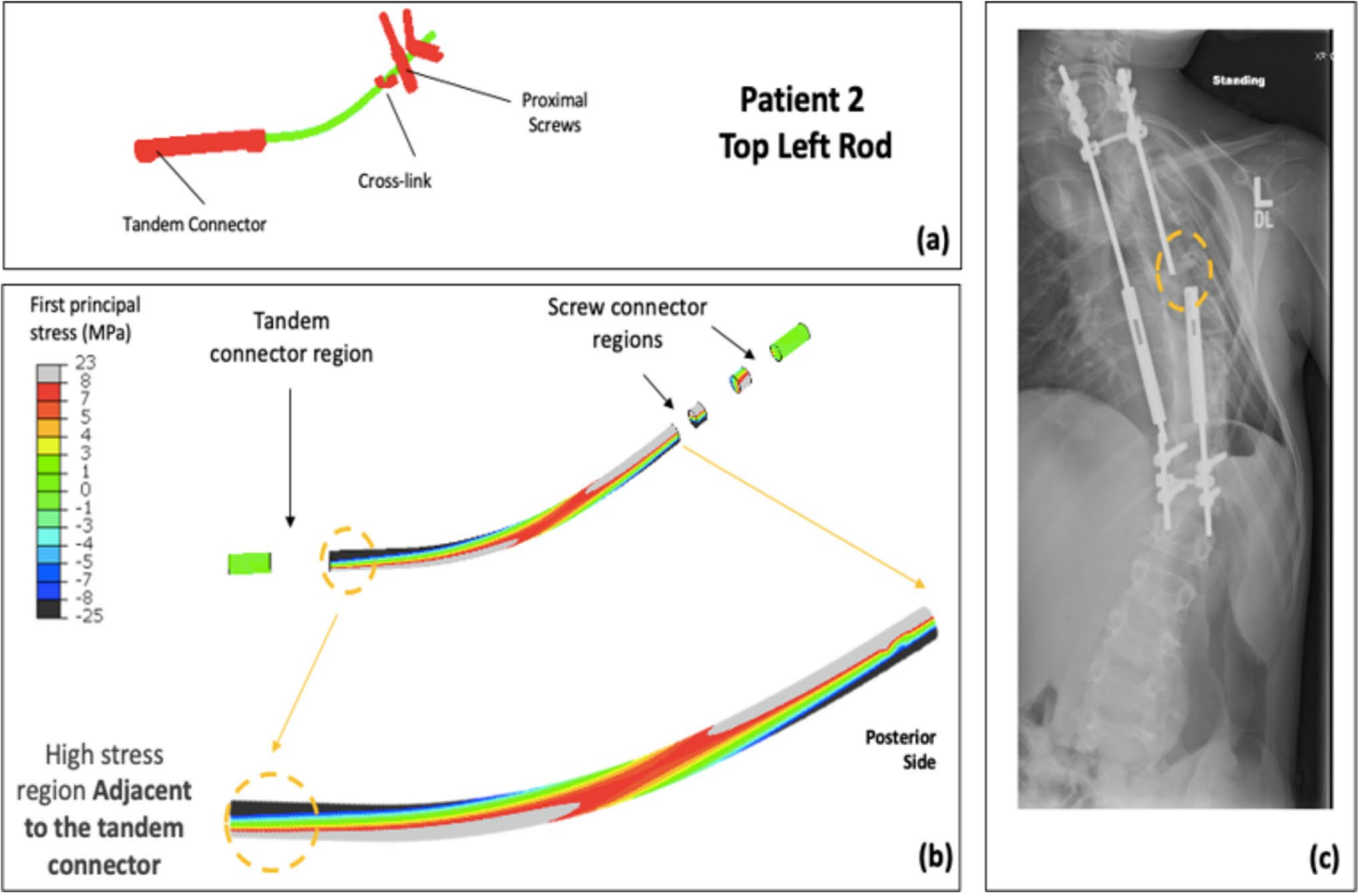
Patient 2 FEA stress distribution plot of top left rod (convex side) compared to the corresponding rod-failure location obtained from retrieval analysis data. **a** FE construct showing top left rod with tandem connector and screws that were used as part of the traditional growing rod construct simulated in the patient-specific FEA model. **b** Stress distribution on top left rod (excluding interacting surfaces). **c** Corresponding patient radiographic image taken from retrieval data to show clinical rod-failure location (encircled) adjacent to tandem connector

**Fig. 6:**
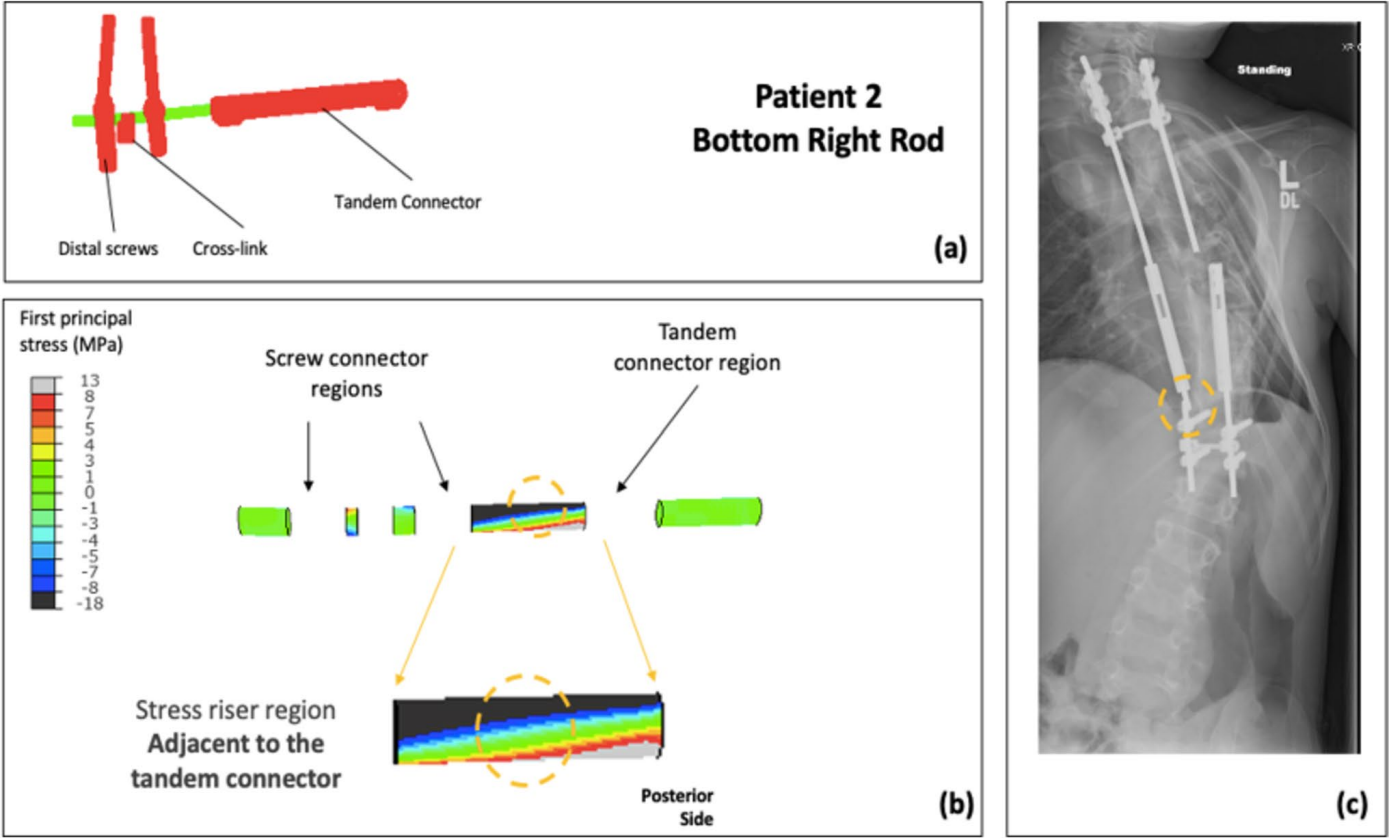
Patient 2 FEA stress distribution plot of bottom right rod (concave side) compared to the corresponding rod-failure location obtained from retrieval analysis data. **a** FE construct showing bottom right rod with tandem connector and screws that were used as part of the traditional growing rod construct simulated in the patient-specific FEA model. **b** Stress distribution on bottom right rod (excluding interacting surfaces). **c** Corresponding patient radiographic image taken from retrieval data to show clinical rod-failure location (encircled) adjacent to tandem connector

**Fig. 7:**
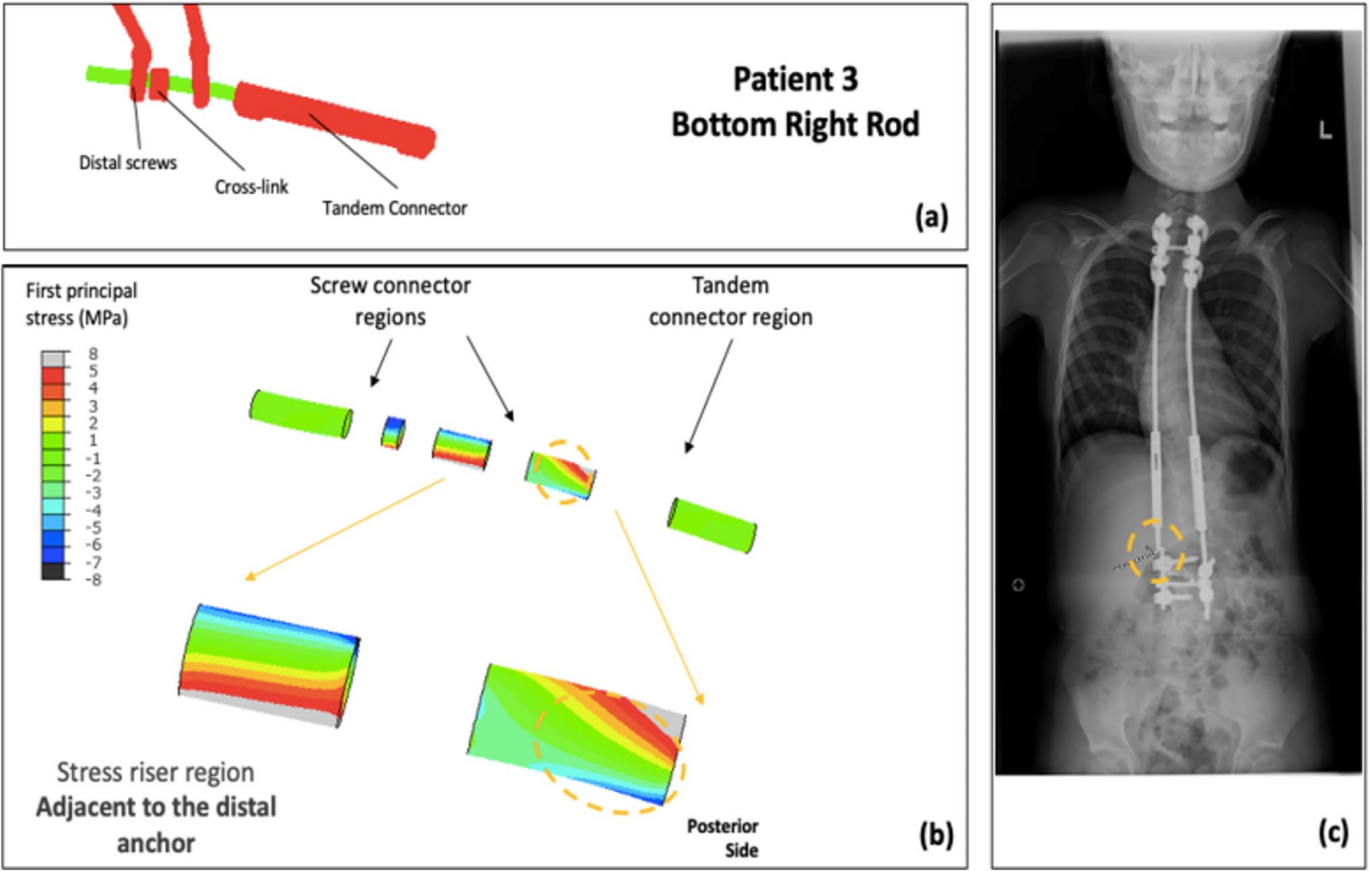
Patient 3 FEA stress distribution plot of bottom right rod (convex side) compared to the corresponding rod-failure location obtained from retrieval analysis data. **a** FE construct showing bottom right rod with tandem connector and screws that were used as part of the traditional growing rod construct simulated in the patient-specific FEA model. **b** Stress distribution on bottom right rod (excluding interacting surfaces). **c** Corresponding patient radiographic image taken from retrieval data to show clinical rod-failure location (encircled) adjacent to distal anchor

The spatial distribution of FEA stresses for each of the rods compared to the retrieval data was analyzed (Figs. 4–7). The posterior surface was examined, because the fracture initiation sites identified in previous retrieval analyses were on the posterior surface [23]. For Patient 1, the bottom left rod failed. The high-stress region in the mid-construct region observed on the bottom left rod in the FEA model matched the failure location from the retrieval data (Fig. 4). Patient 2 had two fracture locations: the top left and the bottom right rod. For the top left rod, the high-stress region observed adjacent to the tandem connector in the FEA model matched the retrieval data (Fig. 5). Furthermore, the fracture location for the bottom right rod matched the high-stress location in the FEA model (Fig. 6). Finally, the identified high-stress region in the FEA model matched the retrieval data for Patient 3 (adjacent to distal anchor, Fig. 7). The stress distribution data shown in Figs. 4–7 correspond to the post-operative radiographs at the last follow-up before rod breakage.

The magnitude of principal stress at the failure location in each of the localized rod regions (identified from the qualitative study) was within 30% of the maximum principal stress on the identified rod for all patients except patient 1 (Table 4). Similarly, the spatial location of these maximum principal stress regions on each of the rods showed a close correlation (within 10 mm—length of the growing rod that interfaces with one pedicle screw) when compared to the radiographically measured location of rod failure for all patients except patient 1 (Table 5).

**Table 4:** Comparison of maximum stress near fracture location to global maximum stress in rod. The maximum principal stress in the entire rod was compared to the localized maximum principal stress near the fracture location identified by the qualitative study

	Rod	Location of max stress (MPa)	Global max stress (MPa)	% Difference
Patient 1	Bottom left	86	206	58
Patient 2	Bottom right	9	13	31
	Top left	17	23	26
Patient 3	Bottom left	7	8	13

**Table 5:** Comparison of maximum stress location in the FEA model with the clinical fracture locations. The FE model distances were calculated using the bottom of the nearest tandem connector (except for patient 2 where the top end of rod was used) as an endpoint. The radiographic calculations were carried out using Surgimap with the same endpoint markers as in the FEA model calculations for consistency

	Rod	FE model	Surgimap
		Maximum stress location (mm)	Fracture location (mm)
Patient 1	Bottom left	10.4	119.0
Patient 2	Bottom right	1.9	10.8
	Top left	97.6	108.1
Patient 3	Bottom left	2.0	12.1

## Discussion

Rod fracture is a common complication with high incidence rates, especially in growing rod implants used for EOS patients [4–9]. A recent proof-of-concept study developed a patient-specific FEA framework to identify and validate the high-stress regions of traditional growing rods against their fracture location obtained from clinical registry data [22, 23]. This study helped establish that clinically observed fracture regions had high localized stress values; two out of the three high-stress regions matched the data from the retrieval analysis conducted by Hill et al. [23]. The results of the current study reinforce the validity of this framework, where the high-stress regions on the FEA growing rods match the fracture locations in the clinical data for all three patients.

Location-wise, the most frequently observed failure locations were adjacent to the distal anchor and adjacent to the tandem connector. Additionally, the results also suggest that the distal region might be more susceptible to fracture. The higher incidence of fractures at these specific locations matches the retrospective clinical observations of a larger study conducted by Hill et al. [23]. The quantitative data are largely consistent (exception in patient 3) in the magnitude and location of maximum stress regions when compared to the clinical data (Tables 4 and 5). As seen in patient 3, there is a significant difference in the stress magnitude in the localized high-stress region when compared to the maximum stress on the fractured rod (Fig. 4, difference of 58%). This actual location of the maximum stress is closer to the tandem connector, as opposed to being adjacent to the distal screw. This could be attributed to the fact that there were two high-stress regions at the proximal and distal ends of the long rod (bottom left rod, Fig. 4). The authors posit that this difference could be attributed to the intra-operative procedures such as notching on the rods, along with other biomechanical effects such as residual stresses due to spinal rod contouring and rod-screw interconnection assembly, which are not included in the scope of the study. [30–32].

Here, it is important to highlight that the authors used static simulations (i.e., no modeling of local damage or damage accumulation) for the purpose of this FEA study. This assumption of correlating stress concentrations from static simulations to fatigue fracture location has been confirmed to be reasonable in other biomechanical studies and employed in ASTM FEA standards as well (e.g., ASTM F2996, F3161, and F3334) [31, 33–36]. These fractures could also have occurred due to loading conditions specific to each patient such as trauma or common physical activities that cause significant flexion or extension in the spine. These simplified simulation methods, therefore, were able to successfully match the high-stress regions in the FEA to the clinical failure locations. This also follows previously published biomechanical data which showed factors like patient weight and distraction loading significantly contributed to generation of high stresses on the rods [37, 38]. Therefore, this patient-specific FEA framework has the potential to help identify high-risk areas and examine the underlying cause of potential failures, with the flexibility to be tailored to different loading regimes in different clinical applications. To our knowledge, the present study is the first in the field of early onset scoliosis to combine patient-specific FEA with clinical registry data to biomechanically evaluate potential rod failure in traditional growing rods.

The current study has some limitations, one being the limited cohort size of the study. This multi-patient study, however, builds on the previous study (limited to one patient compared to a pool of clinical data) and effectively validates the framework for future investigations of failure biomechanics in EOS. Another limitation is the potential error introduced due to a single operator using Surgimap software to obtain the radiographic measurements. Future efforts will quantify operator error through an uncertainty analysis for the multi-patient data. Similarly, another source of potential error is the use of the same material properties across all patients sourced from published literature. However, availability and extraction of patient-specific material properties for a retrospective study is very challenging. Given this limitation, validation studies focusing on patient-specific-FE models have a comparable sample size, in terms of datapoints use [29, 36, 38, 39]. Literature has shown this to mainly affect the range of motion of the spine, which would primarily affect the magnitude of the stresses recorded on the rods [40]. Furthermore, the magnitude of stresses reported in this study seems to be on the lower spectrum compared to the yield stress of the rod material. This is possibly due to the static loading assumption. However, given the scope of the current study, where the focus is on evaluating the distribution of stress on the rods to identify relatively high-stress concentration regions, evaluation of the magnitude of stress becomes less relevant. In addition, these stresses may further intensify with fatigue loading, vary among patients, and may even increase with consecutive distractions for each individual (due to changes in spinal flexibility and/or autofusion) [41].

Overall, this study aids in understanding implant failure from a biomechanical perspective. This patient-specific finite element modeling framework showed a decent correlation to clinical results when examining three EOS patients. Therefore, the computational modeling framework adopted in this study has clinical relevance, since we were successful in validation of this patient-specific in-silico modeling framework; ratified via retrospective clinical data to predict rod-failure locations. With further refinement, this approach may provide specific information that would be valuable to the surgeons/end users about the failure-prone regions on implants to supplement surgical planning. This refinement can be achieved in future studies by performing uncertainty quantification and incorporating more patients retrospectively and potentially prospective as well. This future work would also include analysis based on a variety of device characteristics. Additionally, the results strengthen the finding of the clinical retrieval analysis from a biomechanical perspective and may help device manufacturers optimize implant designs for future cases.

## Conclusion

Overall, this study helps further validate the patient-specific finite element modeling framework. FEA stress concentrations spatially correlated to the analogous retrieval data for all patients. The authors believe that this framework may aid in predicting traditional growing rod failures by providing specific information that would be valuable to the surgeons/end users about the failure-prone regions on implants to supplement surgical planning. Furthermore, this biomechanical analysis may aid manufacturers in device design and development, to optimize the implant design for reducing rod failures. The study also elucidates the potential of utilizing clinical/patient registry data as a validation comparator to help the incumbent state of spinal treatment by providing biomechanical reasoning on traditional growing rod failure which remains a question in the treatment of early onset scoliosis.
